# Comparative analysis of different artificial neural networks for predicting and optimizing *in vitro* seed germination and sterilization of petunia

**DOI:** 10.1371/journal.pone.0285657

**Published:** 2023-05-11

**Authors:** Hamed Rezaei, Asghar Mirzaie-asl, Mohammad Reza Abdollahi, Masoud Tohidfar

**Affiliations:** 1 Department of Plant Biotechnology, Faculty of Agriculture, Bu-Ali Sina University, Hamedan, Iran; 2 Department of Agronomy and Plant Breeding, Faculty of Agriculture, Bu-Ali Sina University, Hamedan, Iran; 3 Department of Plant Biotechnology, Faculty of Life Science and Biotechnology, Shahid Beheshti University, Tehran, Iran; University of Brescia: Universita degli Studi di Brescia, ITALY

## Abstract

The process of optimizing *in vitro* seed sterilization and germination is a complicated task since this process is influenced by interactions of many factors (e.g., genotype, disinfectants, pH of the media, temperature, light, immersion time). This study investigated the role of various types and concentrations of disinfectants (i.e., NaOCl, Ca(ClO)_2_, HgCl_2_, H_2_O_2_, NWCN-Fe, MWCNT) as well as immersion time in successful *in vitro* seed sterilization and germination of petunia. Also, the utility of three artificial neural networks (ANNs) (e.g., multilayer perceptron (MLP), radial basis function (RBF), and generalized regression neural network (GRNN)) as modeling tools were evaluated to analyze the effect of disinfectants and immersion time on *in vitro* seed sterilization and germination. Moreover, non‑dominated sorting genetic algorithm‑II (NSGA‑II) was employed for optimizing the selected prediction model. The GRNN algorithm displayed superior predictive accuracy in comparison to MLP and RBF models. Also, the results showed that NSGA‑II can be considered as a reliable multi-objective optimization algorithm for finding the optimal level of disinfectants and immersion time to simultaneously minimize contamination rate and maximize germination percentage. Generally, GRNN-NSGA-II as an up-to-date and reliable computational tool can be applied in future plant *in vitro* culture studies.

## Introduction

Petunia (*Petunia hybrida*) as an economically super ornamental plant has been cultured throughout the world because of its high diversity in color and morphological traits [1]. Conventionally, petunia is propagated through seed as a sexual method, and through cuttings as non-sexual or vegetative method [2]. However, both conventional propagation methods present limitations that cause the plant propagation to be difficult, vegetative methods due to the time-consuming and season-depending production [3], intensive labor requirements as well as the death of a large number of cutting-derived plants, and sexual methods due to the high heterozygosis [4]. In addition, seedlings propagated by mentioned methods are strongly affected by pest infestation and diseases [5]. Consequently, novel approaches must be essayed for the large-scale cultivation of petunia plants. In this regard, the development of plant tissue culture techniques can be considered as one of the desirable methodologies for the micropropagation and biotechnological exploitation of many plant species with added-value properties [6, 7]. Besides this, *in vitro* culture techniques can be utilized for the rapid production of true-to-type plants [7–15], developmental biology [16–21], secondary metabolite production [22–25], and bioenergy production [26]. Several studies have previously attempted on applying *in vitro* culture techniques to propagate the different cultivars of petunia [2, 4, 5]. The findings of these studies have clearly emphasized that the petunia *in vitro* culture optimization is a genotype-dependent process [2]. Indeed, it found that the best micropropagation obtained for one cultivar may be different from the results attained for other petunia cultivars, which is probably due to differences in their genetic background [2]. The importance of *in vitro* seed germination of petunia lies in its ability to produce a large number of genetically identical plants in a short period of time. This is particularly useful in the production of ornamental plants, where uniformity in plant size and flower color is desirable [27]. *In vitro* seed germination also allows for the production of disease-free plants, as the sterilization process eliminates any fungal or bacterial pathogens that may be present in the seed coat [28]. This is especially important in the production of plants for export, as many countries have strict regulations on the import of plant material that may carry diseases [28]. Moreover, successful *in vitro* seed germination is not only important for studying factors affecting cultivation conditions, but also crucial for obtaining juvenile tissue as a potential explant for different *in vitro* procedures (e. g. gene transformation, callus culture, secondary metabolite and biofuel production) and for screening stress-tolerant genotypes [29].

On the other hand, it has not yet been easy to micropropagation of some economically important plant species such as petunia due to the emergence of some problems during the sterilization as the first and foremost step [30]. Indeed, explants such as seeds collected from the field and greenhouse are contaminated with a broad spectrum of microbes such as bacteria, yeasts, mites, virus, and fungi [28, 31–33]. Therefore, the disinfection of explants can be considered as the most important and first step in successful *in vitro* propagation [34, 35]. The type and concentration of disinfectants (such as ethanol, mercuric chloride (HgCl_2_), bromine water, antibiotics, hydrogen peroxide (H_2_O_2_), nano-silver (NS), silver nitrate (AgNO_3_), sodium hypochlorite (NaOCl), and calcium hypochlorite [Ca(ClO)_2_]) and immersion time are the most important factors during in vitro disinfection [34–36]. Furthermore, optimizing *in vitro* sterilization plays a pivotal role in preventing phenolic browning and contamination of the explants [36–38].

It has been well-documented that the successful explant decontamination is affected by many factors such as culture conditions, plant materials, type and concentration of disinfectants, pH of the media, temperature, light and immersion time [28, 35, 36]. Additionally, it should be noticed that the responses of different petunia cultivars to *in vitro* propagation might be significantly varied depending on the interacting the mentioned factors during *in vitro* sterilization, even in closely related species [2]. Therefore, optimizing the special *in vitro* culture condition is necessary for each cultivar.

*In vitro* micropropagation is a multifactorial and complex biological process, because it is influenced by genotypes/cultivar and many keys interacting factors that would be required for optimizing this mentioned process [39–42]. Commonly, revealing all the information encrypted over the large datasets of biological interactions by traditional statistical techniques is a highly challenging task, particularly when datasets are nonlinear in nature, complex, noisy and ambiguous such as in multifactorial processes of *in vitro* culture [39]. For this purpose, advanced computer-based technologies such as machine learning (ML) tools are capable to analyze and predict complex and multivariate datasets [43]. Advantageously, the use of ML approaches provides the ability to learn autonomously and transform data into useful information without being humanly programmed [44]. Among the different algorithm-based ML tools, artificial neural networks (ANNs) have been proposed as the most powerful ML tools for modeling and predicting complex processes [45–47]. Multilayer perceptron (MLP), generalized regression neural network (GRNN), and radial basis function (RBF) are three popular interpolation neural network models [39, 40]. MLP and other ANNs are made up of a large number of neurons and each neuron has its weight [35]. Indeed, the number of neurons in the hidden layers plays a significant role in the MLP’s design [43]. Although RBF and MLP have similar functions, RBF has a high ability to be used in more than one dimension, in contrast to the MLP [48]. RBF is claimed to be effective for predictions that use approximation multivariate functions wherever suitable characteristics are included [49]. GRNN as another statistic ANN tool belongs to a category of RBF [49]. Simplicity of network structure, very fast network training speed, strong non-linear mapping capability, ease of implementation, high fault tolerance, and high robustness in the solution of complex problems are excellent features of GRNN [50]. Recent studies have reported the good performance and superior predictive accuracy of ANNs tools over traditional statistics for predicting and optimizing *in vitro* culture systems of different plant species such as chrysanthemum [35, 51–54], passion fruit [50], *Prunus* rootstock [55–57], tomato [58], chickpea [59, 60], wheat [49], cannabis [28, 29, 61–64], and ajowan [65]. In addition, the combination of ANNs with an evolutionary optimization algorithm as a superior and reliable computational method confers advantages to predict critical factors that impact plant growth parameters in *in vitro* culture systems [39]. The non-dominated sorting genetic algorithm-II (NSGA-II), known as a search algorithm for optimizing multi-objective problems, is a powerful tool for solving various problems and optimizing and predicting complex processes easier [39]. Also, it provides a simplistic interpretation of results, simultaneously [48]. As an example, among applications of ML algorithms for *in vitro* seed germination in cannabis, Hesami *et al*., [29] employed and compared three models (i.e., MLP, GRNN, and RBF) in combination with the NSGA-II algorithm for predicting and optimizing the effect of culture media and carbon sources and concluded that GRNN-NSGA-II algorithm had good performance for this purpose.

According to our knowledge, the application of ANN algorithms as a new strategy for modeling and predicting *in vitro* culture of petunia is still unexplored. The overall objective of this investigation is to evaluate the effects of different concentrations of disinfectants on *in vitro* seed sterilization and germination of petunia by using three most commonly ANN algorithms including MLP, GRNN, and RBF to compare their ability to model and optimizing of *in vitro* seed sterilization and germination of petunia and employing NSGA-II to predict the best effective level of disinfectants and immersion time on *in vitro* seed sterilization and germination of petunia.

## Materials and methods

### Plant material and *in vitro* culture establishment

In this study, ‘Red Fire chief’ petunia seeds with 95% viability were selected for in vitro sterilization study. Explants were pre-sterilized with a liquid soap solution and 6–7 times washed with tap water. In the next step, the explants were surface sterilized with 70% aqueous ethanol for 30 s and were washed two times with sterilized distilled water under a laminar airflow chamber condition. After applying sterilization treatments, the seeds were washed with sterilized distilled water for 5 min in three times. then, the explants were immediately cultured in the caped glass containing 25 mL of one-tenth strength Murashige and Skoog [66] as a basal medium supplemented with 0.7% agar and 3% sucrose. The pH of the medium was adjusted to 5.7–5.8 prior to autoclaving at 121°C for 15 min. The cultures were incubated in 16 h-photoperiod with a light intensity of 55 μmol m^−2^ s^−1^ in the growth chamber at 25 ± 3°C.

### Experimental design and data analysis

The effect of various concentrations and types of disinfectants at different immersion time on contamination rate and seed germination percentage was studied after three weeks from culturing according to the following treatments.

1. 0, 0.5, 1, 1.5, and 2% NaOCl at 5, 10, and 15 min immersion time (Table 1).
2. 0, 6, 7, 8, and 9% Ca(ClO)_2_ at 5, 10, and 15 min immersion time (Table 2).
3. 0, 1, 2, 4, and 6% HgCl_2_ at 3, 6, and 12 min immersion time (Table 3).
4. 0,10, 12.5, 15, and 17.5% H_2_O_2_ at 10, 15, and 20 min immersion time (Table 4).
5. 0, 2.5, 7.5, and 10 mg/l NWCN-Fe at 10, 15, and 20 min immersion time (Table 5).
6. 0, 50, 100, 150, and 200 mg/l MWCNT at 5, 10, and 15min immersion time (Table 6).

**Table 1:** Effects of various levels of NaOCl in different immersion time on contamination rate and seed germination percentage of petunia.

NaOCl (%) ×time (min)	Contamination rate (%)	Seed germination percentage (%)
0×5	96.8±0.18	16.6±0.26
0×10	96.8±0.18	16.6±0.26
0×15	96.8±0.18	16.6±0.26
0.5 × 5	54.1 ±0.858	42.7±1.09
0.5×10	42.7±0.69	62.5±0.5
0.5×15	44.7±0.26	62.5±0.7
1×5	73.9±0.58	39.5±0.25
1×10	55.2±0.32	54.1±0.82
1×15	29.1±.42	71.8±.0375
1.5×5	29.1±1.37	69.7±1.11
1.5×10	14.5±0.94	62.5±0.65
1.5×15	16.6±0.59	75±0.86
2×5	16.6±0.60	79±0.62
2×10	15.6±0.61	72.9±0.9
2×15	7.0±0.62	80.2±0.49

Values in each column represent means ± Standard error

**Table 2:** Effects of various levels of Ca(ClO)_2_ in different immersion time on contamination rate and seed germination percentage of petunia.

Ca(ClO)_2_(%)× time (min)	Contamination rate (%)	Seed germination percentage (%)
0×5	96.8±0.18	16.6±0.26
0×10	96.8±0.18	16.6±0.26
0×15	96.8±0.18	16.6±0.26
6×5	5.20±0.49	87.5±0.53
6×10	3.12±0.37	82.29±0.95
6×15	6.25±0.52	58.33±0.32
7×5	0±0.0	76.04±0.35
7×10	4.16±0.5	64.58±0.75
7×15	8.33±0.65	61.45±0.37
8×5	3.12±0.37	79.16±0.56
8×10	4.16±0.5	65.62±1.11
8×15	4.16±0.5	80.2±0.59
9×5	11.45±0.62	72.91±0.49
9×10	4.16±0.5	91.66±0.37
9×15	6.25±0.75	80.20±0.84

Values in each column represent means ± Standard error

**Table 3:** Effects of various levels of HgCl_2_ in different immersion time on contamination rate and seed germination percentage of petunia.

HgCl_2_(%) × time (min)	Contamination rate (%)	Seed germination percentage (%)
0×3	96.8±0.18	16.6±0.26
0×6	96.8±0.18	16.6±0.26
0×12	96.8±0.18	16.6±0.26
1×3	5.2±0.69	67.7±0.49
1×6	9.37±0.54	59.37±0.65
1×12	14.58±0.58	73.99±0.49
2×3	14.58±0.74	57.29±0.59
2×6	14.58±0.75	61.45±0.375
2×12	12.51±0.46	61.45±0.12
4×3	12.51±0.67	63.54±0.0
4×6	13.53±0.41	69.79±0.25
4×12	16.66±0.86	64.58±0
6×3	12.5±0.51	73.95±0.26
6×6	7.29±0.77	61.45±0.26
6×12	10.41±0.67	36.45±0.0

Values in each column represent means ± Standard error

**Table 4:** Effects of various levels of H_2_O_2_ in different immersion time on contamination rate and seed germination percentage of petunia.

H_2_O_2_ (%) ×time(min)	Contamination rate (%)	Seed germination percentage (%)
0×10	96.8±0.18	16.6±0.26
0×15	96.8±0.18	16.6±0.26
0×20	96.8±0.18	16.6±0.26
10×10	31.25±0.59	66.66±0.56
10×15	28.12±0.62	63.54±0.53
10×20	27.08±0.61	61.45±0.41
12.5×10	14.58±0.67	66.66±0.46
12.5×15	15.62±0.63	62.5±0,73
12.5×20	27.08±1.16	55.2±1.08
15×10	6.25±0.49	78.12±0.41
15×15	7.29±0.22	67.7±0.61
15×20	16.66±0.73	67.7±0.58
17.5×10	4.16±0.26	55.2±0.44
17.5×15	1.04±012	62.5±0.42
17.5×20	4.16±0.26	59.37±0.39

Values in each column represent means ± Standard error

**Table 5:** Effects of various levels of NWCN-Fe in different immersion time on contamination rate and seed germination percentage of petunia.

NWCN-Fe(mg/l) ×time(min)	Contamination rate (%)	Seed germination percentage (%)
0×10	96.8±0.18	16.6±0.26
0×15	96.8±0.18	16.6±0.26
0×20	96.8±0.18	16.6±0.26
2.5×10	80.20±0.49	30.20±0.80
2.5×15	75±0.70	33.33±0.96
2.5×20	53.12±1.30	47.91±1.29
5×10	34.37±1.00	60.41±0.70
5×15	43.75±0.81	58.33±0.90
5×20	38.54±0.90	50±0.75
7.5×10	31.25±0.45	55.20±0.53
7.5×15	66.66±0.84	32.29±1.39
7.5×20	55.20±1.22	30.20±1.23
10×10	62.5±1.25	30.20±1.01
10×15	37.5±0.90	62.5±1.11
10×20	61.45±0.56	41.66±1.03

Values in each column represent means ± Standard error

**Table 6:** Effects of various levels of MWCNT in different immersion time on contamination rate and seed germination percentage of petunia.

MWCNT× time(min)	Contamination rate (%)	Seed germination percentage (%)
0×5	96.8±0.18	16.6±0.26
0×10	96.8±0.18	16.6±0.26
0×15	96.8±0.18	16.6±0.26
50×5	48.95±1.18	48.95±0.54
50×10	43.75±0.92	29.16±0.70
50×15	56.25±1.67	30.2±0.82
100×5	60.41±0.75	40.62±0.44
100×10	55.2±1.03	42.7±0.87
100×15	43.75±0.88	38.54±0.75
150×5	45.83±0.75	43.75±0.79
150×10	69.79±0.98	29.16±0.70
150×15	60.41±0.55	33.33±0.65
200×5	79.16±0.77	22.91±0.45
200×10	93.75±0.52	10.41±0.49
200×15	66.66±0.96	32.29±0.83

Values in each column represent means ± Standard error

The experiment was conducted using a completely randomized design (CRD) with the factorial arrangement (total of 76 treatments) with 12 replicates per treatment following with 8 sub-sets (total of 608 datapoint).

### Modeling and optimization

#### ANN modeling

**Fig 1:**
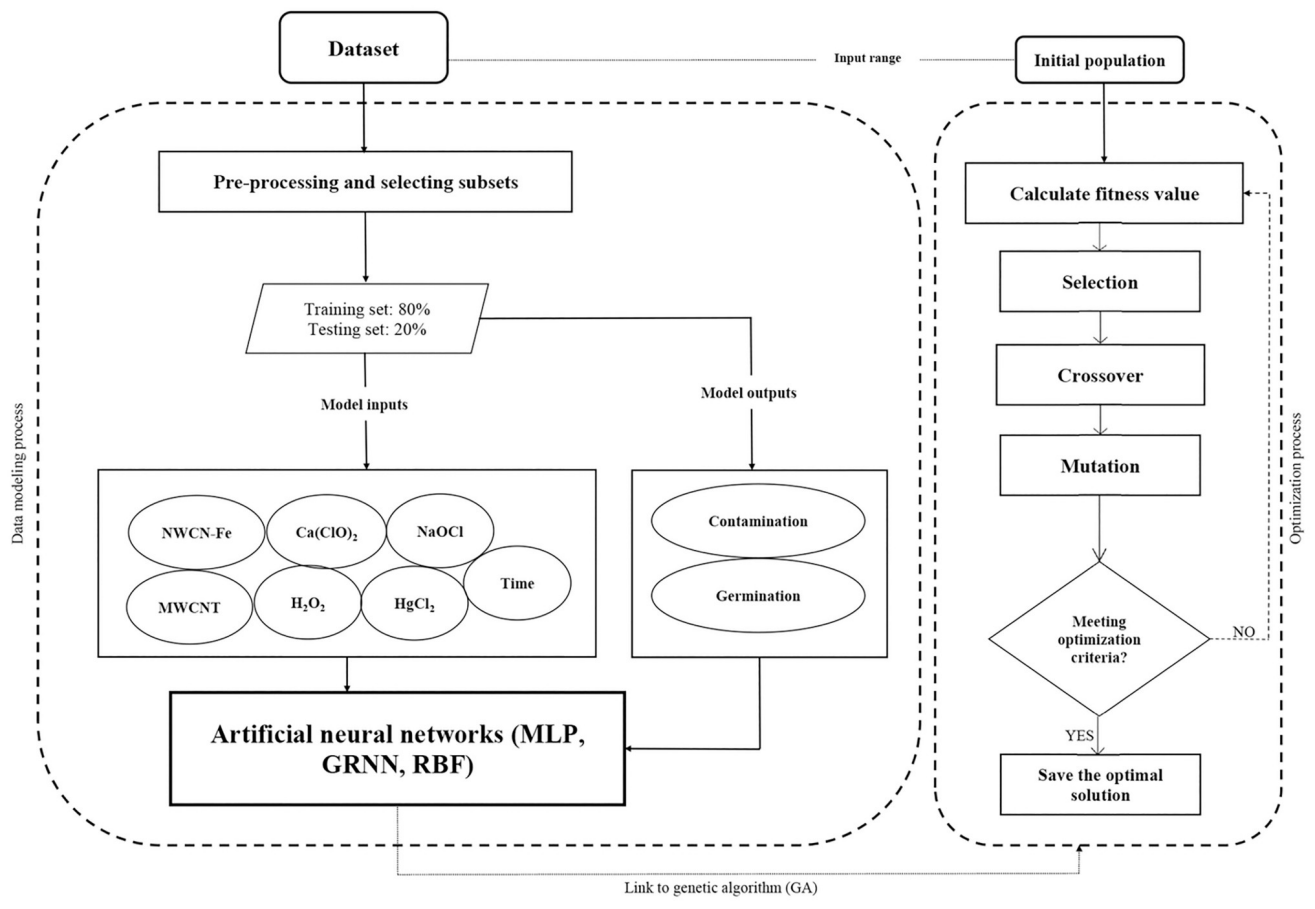
A schematic representation of machine learning methodology in this study.

There is a wide range of ML algorithm tools that help humans to make predictions and interpretation of results by building computational models using datasets as training and testing data. In the current study, three of the most widely used feed-forward ANNs (MLP, RBF, and GRNN) were chosen to model and decipher the effect of immersion time of various disinfectants on *in vitro* seed sterilization and germination of petunia. The ANN models were classified using 7 independent variables including NaOCl, Ca(ClO)_2_, HgCl_2_, H_2_O_2_, NWCN-Fe, MWCNT, and immersion time as inputs and two *in vitro* responses (i.e., contamination rate and seed germination percentage) as outputs. The input data was structured as a matrix with dimensions (608 samples, 7 input). Therefore, the shape of our dataset was (608 samples, 7 input). Finally, the output of the ANNs was a matrix with dimensions (608 samples, 6 input), where each row corresponded to the predicted values of the two output variables for a given input sample. Data standardization was employed prior to applying ANN modeling to normalize and detect outliers in the data for each cultivar; the datasets were standardized between 0 and 1. Then, principal component analysis (PCA) was used to detect outlier data, however, no outlier data was identified. To train and test all three models, the experimental data were randomly divided into 80% and 20% parts, respectively. The structural layout of the ANN algorithm is depicted in Fig 1.

#### Multilayer perceptron (MLP) model

The MLP-based back-propagation algorithm, one of the most commonly ANN methods, consists of three layers: the input layer, one or more hidden layers, and the output layer. For the model construction, the MLP algorithm was applied with 2 hidden layers. The first hidden layers had 128 nodes, while the second hidden layer had 64 nodes. The activation function for hidden and output layers was set to hyperbolic tangent sigmoid function (tansig) and linear function (purelin), respectively. The Levenberg-Marquardt algorithm was employed to adjust the bias and weights in the training set of the network. To find the best topology of the model structure, the optimal number of neurons in the hidden layers was detected based on trial-and-error analysis. Additionally, the error was minimized between every input and output variable according to the following equation:

$$Error=\frac{1}{n}\sum_{n=1}^{n}{\left(O_{i}-P_{i}\right)}^{2}$$ (1)

In which, *O*_*i*_ and *P*_*i*_ display the measured values and predicted value, respectively. *n* is the total number of data.

In an MLP model with *n* inputs and *m* neurons in the hidden layer *P*_*i*_ is obtained from the Eq (2):

$$P_{i}=f\left[\sum_{j=1}^{m}w_{j}.g\left(\sum_{i=1}^{n}w_{ji}x_{i}+w_{j0}\right)+w_{0}\right]$$ (2)

where *m* is the number of neurons in the hidden layer. *x*_*i*_ and *n* represent the *i*^*th*^ input variable and output variables, respectively. *w*_0_ and *w*_*j*0_ display the bias of output neurons and *j*^*th*^ the neuron of the hidden layer. *f* and *g* denote the transfer functions for the output and hidden layer, respectively. *w*_*ji*_ and *w*_*j*_ indicate the weight connecting the *j*^*th*^ the neuron of the hidden layer and the weight linking the neuron of the output layer.

#### Radial basis function (RBF) model

RBF has one hidden layer between an input and an output layer. The input layer had 7 neurons, corresponding to the 7 input features. The hidden had 120 neurons. The output layer had 1 neuron for each output. RBF, like MLP, is an error-back-propagation ANN and also has similar functions to MLP. However, RBF-ANN does not enter raw data into the network and it is only possible that the weights between hidden and output layers are modified via an error signal, thus, the RBF requires a much shorter learning time compared to the MLP [29]. In RBF, the Euclidean distance is measured between the inputs and hidden layer to provide the activation function for each neuron. In the hidden layer neurons, the Gaussian function is defined as a transfer function:

$$\varphi_{j}\left(x\right)=exp\left(\frac{\left\|x-c_{k}\right\|}{\sigma_{j}^{2}}\right)\;j=1,\;2,\;3\ldots .p$$ (3)

where *φ*_*j*_(*x*), *c*_*k*_, *δ*_*i*_, ‖*x* − *c*_*k*_‖, *x*, and *p* represent the activation function, center, and widths of each Gaussian function, Euclidean distance norm, the input vector of the neuron in the hidden layer, and hidden neuron number. Finally, an output neuron creates a weighted sum of the hidden output to give the network’s final output $\left(\hat{Y}\right)$ after the non-linear projection of the inputs.

$$\hat{Y}=\sum_{j=1}^{J}\varphi_{j}w_{j}$$ (4)

where *J* and *w*_*j*_ denote the number of hidden neurons, and the weight between the hidden layer and output layer.

#### Generalized regression neural network (GRNN) model

The GRNN algorithm is another kind of RBF neural network. The main difference between GRNN and classical RBF is that GRNN has a fast-learning speed even when the number of training samples is limited and quickly converges to the best regression surface [21]. This nonlinear neural network tool consists of input, pattern, summation, and output layers. In this study, the architecture of the GRNN model was 7 neurons for input layer, 608 neurons for pattern layer, 1 neuron for summation layer, and 1 neuron for output layer. The input layer is completely entered the pattern layer and the output of each pattern layer neuron is connected to the summation neurons. Two summation neurons are considered in the output layer, S-summation (Ss) and D-summation (Sd) neurons, that the unweight pattern neuron outputs are computed by Sd neuron (Eq 5), and the weighted sum of pattern neuron outputs are determined by the Ss neuron (Eq 6).

$$S_{s}=\sum_{i=1}^{n}exp\;\left(-\frac{{\left(x-x_{i}\right)}^{T}\left(x-x_{i}\right)}{{2\sigma }^{2}}\right)$$ (5)

$$S_{d}=\sum_{i=1}^{n}w_{i}exp\;\left(-\frac{{\left(x-x_{i}\right)}^{T}\left(x-x_{i}\right)}{{2\sigma }^{2}}\right)$$ (6)

where *w*_*i*_ is the interconnection weight of neuron *i* to the summation layer in the pattern layer, *σ* denotes the width parameter, *x* represents the input variable, and *x*_*i*_ is a specific training vector of pattern *i*^*th*^ neuron.

Finally, by dividing the summation layer outputs, the output layer computes the output *Y*, as the mean of all the weighted observed output data, according to the following equation:

$$Y=\frac{S_{d}}{S_{s}}$$ (7)

### Model evaluation

To assess and compare the accuracy and performance of the ANN algorithms for predicting the proliferation of pomegranate, the coefficient of determination (R^2^) [Eq (8)], root mean square error (RMSE) [Eq (9)], and mean bias error (MBE) [Eq (10)] were applied.

$$R^{2}=\;1-\frac{\sum_{i=1}^{n}{\left(y_{i}-{\hat{y}}_{i}\right)}^{2}}{\sum_{i=1}^{n}{\left(y_{i}-{\bar{y}}_{i}\right)}^{2}}$$ (8)

$$RMSE=\sqrt{\left(\sum_{i=1}^{n}{\left(y_{i}-{\hat{y}}_{i}\right)}^{2}\right)/n}$$ (9)

$$MBE=1/n\sum_{i=1}^{n}\left(y_{i}-{\hat{y}}_{i}\right)$$ (10)

Where ***y***_***i***_ is the value of predicted datasets, ${\hat{y}}_{i}$ is the value of observed datasets, and *n* is the number of data.

### Sensitivity analyses

Sensitivity analysis is a powerful tool used in plant tissue culture to investigate the effects of changes in input variables on the output of a system or model [55]. It is used to identify the most critical input variables that have the greatest impact on the system’s behavior or performance [67]. Sensitivity analysis can be performed using a range of techniques, including one-factor-at-a-time, Morris screening, variance-based methods, and Sobol’ indices. These methods can be applied to both deterministic and stochastic models, and can help to identify sources of uncertainty and improve model accuracy [67].

To determine the effect of the inputs on output variables (dependent variables) as well as to find the most dominant parameters and their degree of importance on the network outputs, a sensitivity analysis was evaluated using the criteria as follows:

- The variable sensitivity error (VSE): displays the overall performance (RMSE) of the model when a particular input variable is removed.
- Variable sensitivity ratio (VSR): displays the correlation between the error of the developed model (RMSE) and VSE in the case that all variables are available.

### Optimization of ANN model via NSGA-II

The Non-dominated Sorting Genetic Algorithm II (NSGA-II) is a powerful multi-objective optimization algorithm that is widely used in plant tissue culture studies [39]. It operates on a population of candidate solutions and uses a fitness function to evaluate the quality of each solution with respect to multiple conflicting objectives [68]. NSGA-II employs a non-dominated sorting procedure that assigns a ranking to each solution based on its Pareto dominance, and uses this ranking to generate a new population of solutions through selection, crossover, and mutation operators [43]. The algorithm also uses a crowding distance measure to ensure that the population is diverse and evenly distributed across the Pareto front. NSGA-II has demonstrated its effectiveness in solving complex optimization problems and has become one of the most popular algorithms in the field of multi-objective optimization [39].

In the current study, the best ANN algorithm as the fitness function was introduced to NSGA-II to find the best combination of inputs (immersion time, NaOCl, Ca(ClO)_2_, HgCl_2_, H_2_O_2_, NWCN-Fe, MWCNT) for achieving maximal *in vitro* seed germination and minimal contamination (Fig 1). In this study, based on natural selection, several parameters were used to create the best optimization of NSGA-II during the optimization process, as follows: the creation of the initial population as the first step of the NSGA-II process which all the chromosomes are constructed in this step. The tournament selection method was chosen to select an elite population for crossover. The crossover function was considered to create the next generation of chromosomes and two-point crossover as the most well-known crossover fraction evaluated in this research. After that, the mutation was applied to reduce local minima in the population, as it can create random variations in chromosomes and reduce the possibility of having similar chromosomes [20, 30]. To improve fitness function during the optimization process, the optimal values of operators (crossover rate, maximum generation, initial population, and mutation rate) were regulated by trial and error. In the current study, crossover rate, maximum generation, initial population, and mutation rate were as 70%, 800, 85, and 0.01, respectively. The ideal point of pareto was chosen such that contamination rate and seed germination percentage became the minimum and maximum, respectively. In other words, a point in the pareto front was considered as the solution such that $\sqrt{{\left(contamination\;rate\;-m\right)}^{2}+{\left(seed\;germination\;percentage-n\right)}^{2}}$ (Eq 11) was minimal; where *m* and *n* are the minimum and the maximum contamination rate and seed germination percentage in observed data, respectively.

All mathematical codes [69] for evaluation of ANN models and ANN-NSGA-II algorithm were performed using Matlab software [70].

### Validation experiments

In order to approve the efficiency of the model, the obtained results from GRNN-NSGA-II were experimentally tested in the lab. The validation experiment was done based on the CRD with three replications and each replication consisted of ten seeds.

## Results

### The effects of different disinfectants and immersion time on *in vitro* seed sterilization and germination

The seeds had various responses to different types and concentrations of disinfectants and immersion time. Contamination was described as following which occurred during the first and second weeks after sterilization treatments.

*I) Effects of different concentrations of NaOCl and immersion time on disinfection parameters*

The application of 2% NaOCl for 15 min and 5 min resulted in the highest percentage of seed germination (80.20% and 79.62%, respectively), while the lowest percentage of seed germination (16.6%) was obtained from control (without NaOCl) treatments. Also, the lowest (7%) and the highest (96.8%) percentage of contamination were observed in 2% NaOCl for 15 min and control treatments, respectively (Table1).

*II) Effects of different concentrations of Ca(ClO)*_*2*_ *and immersion time on disinfection parameters*

The application of 9% Ca(ClO)_2_ for 10 min and 6% Ca(ClO)_2_ for 5 min resulted in the highest percentage of seed germination (94.66 and 87.5%, respectively), while the lowest percentage of seed germination (16.6%) was detected in control (without Ca(ClO)_2_) treatments. Also, the lowest (4.1%) and the highest (96.8%) percentage of contamination were observed in 9% Ca(ClO)_2_ for 10 min and control treatments, respectively (Table2).

*III) Effects of different concentrations of HgCl*_*2*_ *and immersion time on disinfection parameters*

The highest percentage of seed germination (73%) was detected in 1% HgCl_2_ for 12 min and 6% HgCl_2_ for 3 min, while control treatments resulted in the lowest percentage of seed germination (16.6%). Also, 6% HgCl_2_ for 6 min and control treatments led to the lowest (7.3%) and the highest (96.8%) contamination rate, respectively (Table 3).

*IV) Effects of different concentrations of H*_*2*_*O*_*2*_ *and immersion time on disinfection parameters*

The highest percentage of seed germination (78.12%) was detected in 15% H_2_O_2_ for 10 min immersion time, while control treatments resulted in the lowest percentage of seed germination (16.6%). Also, 17.5% H_2_O_2_ for 15 min and control treatments led to the lowest (1.04%) and the highest (96.8%) contamination rate, respectively (Table 4).

*V) Effects of different concentrations of NWCN-Fe and immersion time on disinfection parameters*

The highest percentage of seed germination (62.5%) was detected in 10 mg/l NWCN-Fe for 15 min immersion time, while control treatments resulted in the lowest percentage of seed germination (16.6%). Also, 7.5 mg/l NWCN-Fe for 10 min and control treatments led to the lowest (31.25%) and the highest (96.8%) contamination rate, respectively (Table 5).

*VI) Effects of different concentrations of MWCNT and immersion time on disinfection parameters*

The application of 50 mg/l MWCNT for 5 min resulted in the highest percentage of seed germination (48.95%), while the lowest percentage of seed germination (10.41%) was detected in 200 mg/l MWCNT for 10 min. Also, the lowest (43.75%) and the highest (96.8%) percentage of contamination were observed in 50 and 100 mg/l MWCNT for 10 min and control treatments, respectively (Table 6).

### Comparison of artificial neural networks performance

**Fig 2:**
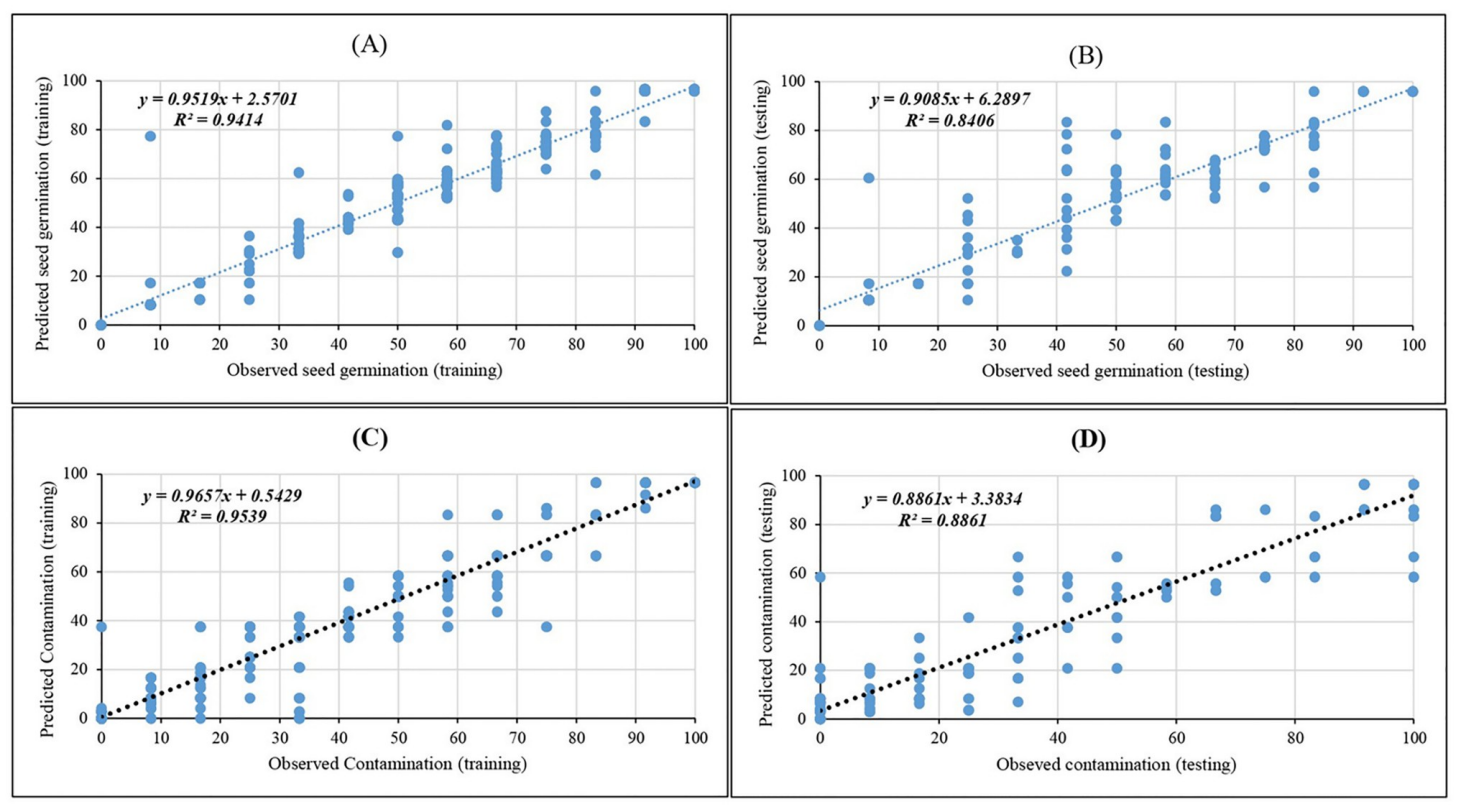
Scatter plot of observed vs. predicted results of studied parameters obtained by GRNN model including (A) Training set and (B) Testing set of seed germination percentage; (C) Training set and (D) Testing set of contamination rate.

In the present study, we used the advantages of three ANN algorithms to build the mathematical models of MLP, RBF, and GRNN. The scatter plots of prediction results of both training and testing the GRNN model are shown in Fig 2. Moreover, in both the training and testing processes of the GRNN model, the regression lines represented the good fit correlation between observed and predicted data for all growth parameters (Fig 2).

The corresponding prediction evaluation indexes of three models are shown in Table 7. With respect to Table 7, both the training and test subset R^2^ between observed (experimental) and predicted values was higher than 79% for all parameters (outputs), which demonstrated that all three ANN models had a good performance and predictability. The GRNN with higher R^2^ and smaller RMSE and MBE values in both training and testing sets was the best algorithm in comparison to three other models for all growth parameters (Table 7). In this regard, the results derived from comparative between evaluation indexes on the measured *in vitro* parameters revealed that the values of the RBF were very close to the GRNN. As can be seen in Table 7, during the training set, R^2^ of GRNN and RBF vs. MLP were 0.954 and 0.944 vs. 0.890 for contamination rate as well as 0.941 and 0.913 vs. 0.846 for seed germination percentage. Also, during the testing set, R^2^ of GRNN and RBF vs. MLP were 0.886 and 0.863 vs. 0.884 for contamination rate as well as 0.841 and 0.800 vs. 0.794 for seed germination percentage (Table 7).

**Table 7:** Comparison statistics of different ANNs including MLP, GRNN, and RBF for modeling and predicting contamination rate and seed germination percentage of petunia.

Model	Measured parameter	Training			Testing		
		R^2^	RMSE	MBE	R^2^	RMSE	MBE
MLP	Contamination rate	0.890	15.948	-2.272	0.844	20.645	-3.565
	Seed germination percentage	0.846	15.383	1.699	0.794	17.219	1.956
GRNN	Contamination rate	0.954	11.632	1.572	0.886	14.836	-2.735
	Seed germination percentage	0.941	10.724	-1.182	0.841	14.026	1.898
RBF	Contamination rate	0.944	13.737	2.024	0.863	18.370	-3.870
	Seed germination percentage	0.913	11.569	-1.237	0.800	15.369	2.870

### Optimization process via non-dominated sorting genetic algorithm-II

**Fig 3:**
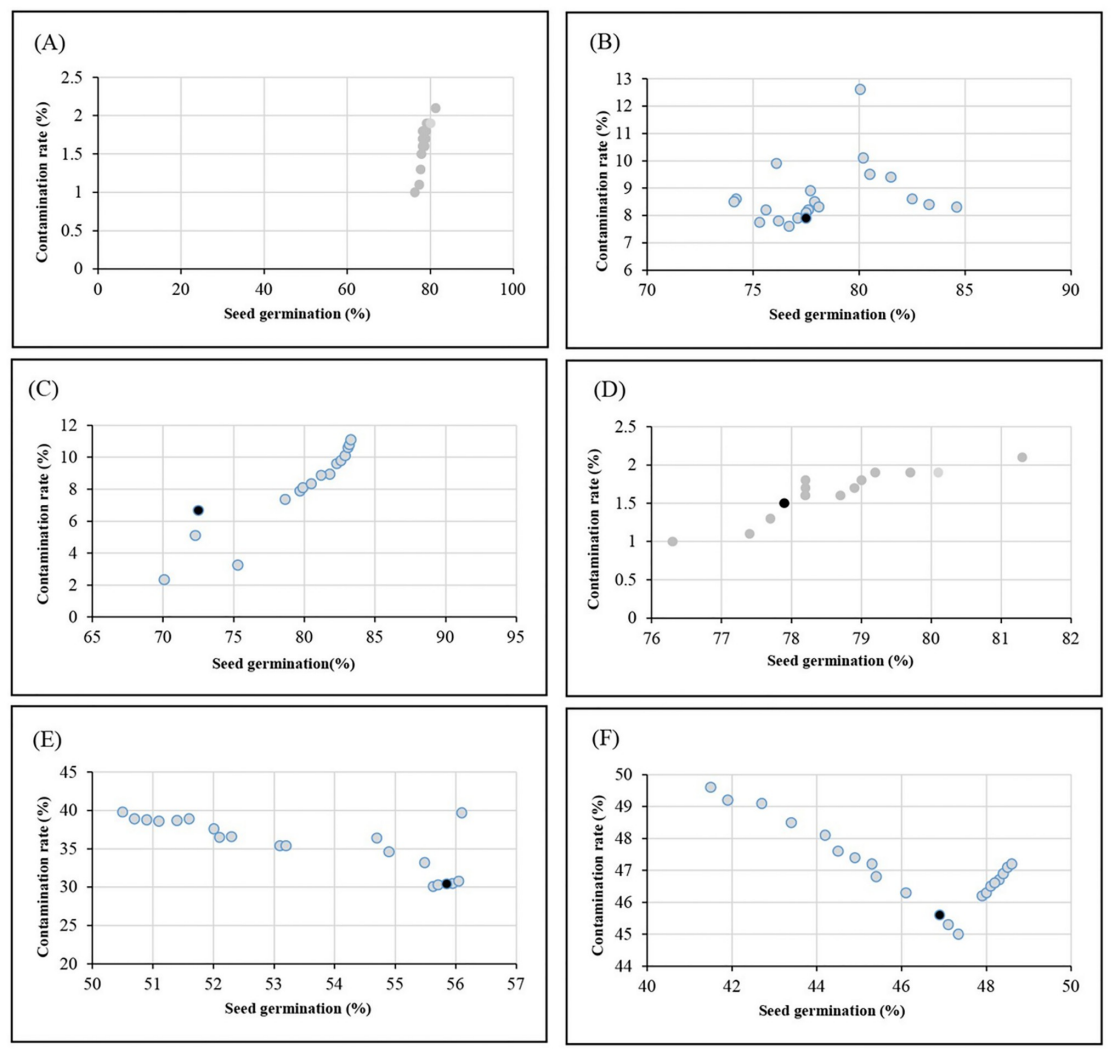
Pareto front reached by NSGAII for achieving the highest seed germination percentage and the least contamination rate of petunia in different disinfection treatments including (A) 8.73% Ca(ClO)_2_ ×13.54 min, (B) 1.89% NaOCl×11.98 min, (C) 5.03 HgCl_2_×4.77 min, (D) 16.35 H_2_O_2_×18.44 min, (E) 8.59 mg/l NWCN-Fe×16.19 min, (F) 35.63 mg/l MWCNT×9.93 min. The black point represents the ideal point.

The NSGA-II algorithm, as multi-objective evolutionary optimization, was linked to the GRNN model which was diagnosed as the most accurate algorithm. GRNN-NSGA-II algorithm was successful to find simultaneously the two *in vitro* parameters (i.e., contamination rate and seed germination percentage) as a function of different levels of immersion time, NaOCl, Ca(ClO)_2_, HgCl_2_, H_2_O_2_, NWCN-Fe, MWCNT. The results of the optimization process were presented in Table 8 and Fig 3. According to Table 8, 8.73% Ca(ClO)_2_ at 13.54 min immersion time can cause 4.27% contamination and 90.63% seed germination (Fig 3A); 1.89% NaOCl at 11.97 min immersion time can result in 8.03% contamination and 76.27% seed germination (Fig 3B); 5.03% HgCl_2_ at 4.77 min immersion time can lead to 8.56% contamination and 72.03% seed germination (Fig 3C); 16.35% H_2_O_2_ at 18.44 min immersion time can cause 1.33% contamination and 77.73% seed germination (Fig 3D); 8.59 mg/l NWCN-Fe at 16.19 min immersion time can result in 33.07% contamination and 56.78% seed germination (Fig 3E); and 35.63 mg/l MWCNT at 9.93 min immersion time can lead to 45.63% contamination and 41.07% seed germination (Fig 3F).

**Table 8:** The results of GRNN-NSGAII to find the optimal level of sterilants and immersion times for contamination rate (%) and seed germination percentage in petunia.

input variable							Contamination rate (%)	Seed germination (%)
NaOCl (%)	Ca(ClO)_2_ (%)	HgCl_2_ (%)	H_2_O_2_ (%)	NWCN-Fe (mg/L)	MWCNT (mg/L)	immersion times (min)		
-	8.73	-	-	-	-	13.54	4.27	90.63
1.89	-	-	-	-	-	11.97	8.03	76.27
-	-	5.03	-	-	-	4.77	7.36	72.03
-	-	-	16.35	-	-	18.44	1.33	77.73
-	-	-	-	8.59	-	16.19	33.07	56.78
-	-	-	-	-	35.63	9.93	45.63	41.07

### Sensitivity analysis

Based on the VSR values, the comparative rank of input variables to characterize their important effect on each output variable can be determined using the entire experimental data. The results of the sensitivity analysis for each output variable were summarized in Table 9. Based on sensitivity analysis, contamination rate was more sensitive to immersion time, followed by NaOCl, HgCl_2_, Ca(ClO)_2_, MWCNT, H_2_O_2_, and NWCN-Fe. Also, as can be seen in Table 9, immersion time was the most important factor for seed germination percentage, followed by NaOCl, Ca(ClO)_2_, H_2_O_2_, HgCl_2_, MWCNT and NWCN-Fe, respectively. This suggested that immersion time and NaOCl can significantly influence both *in vitro* parameters (i.e., contamination rate and seed germination percentage). Sensitivity analysis clearly revealed that the highest VSR in all *in vitro* parameters belongs to the immersion time. Therefore, it can be concluded that the immersion time has a key role in the successful sterilization of petunia (Table 9).

**Table 9:** The results of sensitivity analysis to find the importance of each input for contamination rate (%) and seed germination percentage in petunia.

Output	Item	NaOCl	Ca(ClO)_2_	HgCl_2_	H_2_O_2_	NWCN-Fe	MWCNT	Immersion times
Contamination rate	VSR	3.63	2.76	2.96	1.36	1.14	1.83	3.73
	Rank	2	4	3	6	7	5	1
Seed germination percentage	VSR	1.94	1.72	1.11	1.36	1.01	0.80	2.07
	Rank	2	3	5	4	6	7	1

### Validation experiment

According to the validation experiment, the differences between biological validation data and predicted data via GRNN-NSGA-II were not significant (Table 10). Indeed, the optimized level of disinfectants and immersion time led to an acceptable seed germination percentage with a low contamination rate. Therefore, GRNN-NSGA-II as an up-to-date and reliable computational tool can be applied in future plant in vitro culture studies.

**Table 10:** Experimental validation of the predicted-optimized results using GRNN-NSGA-II for contamination rate and seed germination percentage of petunia.

Treatments	Contamination rate (%)	Seed germination percentage (%)
8.73% Ca(ClO)_2_ ×13.54 min	6.67	93.33
1.89% NaOCl×11.98 min	10	76.66
5.03 HgCl_2_×4.77 min	6.66	73.33
16.35 H_2_O_2_×18.44 min	3.33	76.66
8.59 mg/l NWCN-Fe×16.19 min	33.33	56.66
35.63 mg/l MWCNT×9.93 min	43.33	40

## Discussion

Successful in *in vitro* sterilization protocol of petunia is one of the main challenges on *in vitro* petunia cultures due to the death of the seeds as a result of the high concentration of disinfectants [2]. Using different parts of the greenhouse- and/or field-grown plants as potential explants for *in vitro* propagation causes the major challenges due to the wide range of contaminations such as filamentous, yeasts, fungi, viruses, and bacteria [33, 35]. Therefore, successfulness in plant tissue culture is directly dependent on controlling infections and contaminations [71, 72]. Moreover, each plant and explant can distinctively react to a particular disinfectant [36]. Therefore, the explants should be completely sterilized before culturing them into the culture media [28]. Although different sterilants and immersion time can be employed to sterilize the explants, each explant needs a particular sterilization protocol [35, 71].

In the present study, different disinfectants at various immersion time were used for *in vitro* seed sterilization and germination of petunia. According to our results, the seeds had various responses to different treatments. However, some disinfectants exhibited better effects on controlling contamination and germinating seeds. Our results showed that calcium hypochlorite caused the significant decline in the contamination rate. Several studies [36, 73–75] have effectively controlled contamination by using Ca(ClO)_2_ solution. In accordance with our results, Hesami *et al*., [35] showed that using 10% Ca(ClO)_2_ for 5, 10, and 15 min resulted in controlling contamination (no infection). Although Ca(ClO)_2_ was more effective than NaClO in controlling contamination during our study, NaClO was commonly more used for sterilizing explants in different studies [36]. Higher efficiency of Ca(ClO)_2_ than NaClO may be due to its more chlorine level [30].

Based on our findings, NaClO not only caused to control contamination, but also resulted in acceptable seed germination. Also, our results showed an increase in the level of NaClO and immersion time resulted in the highest percentage of seed germination (80.2%) with low contamination (7%). In line with our results, Miyoshi and Mii [76] reported that an increase in the concentration of NaClO caused to better-controlling contamination with acceptable seed germination. Similar results were also reported by Hesami *et al*., [35]. Moreover, several studies [30, 31, 77] have reported the merit points of NaClO in *in vitro* sterilization and seed germination of petunia.

In the current study, the 3% HgCl_2_ at 3 min immersion time caused to the lowest percentage of contamination. Also, the effectiveness of HgCl_2_ was less and more than Ca(ClO)_2_ and NaClO, respectively. In accordance with our findings, Hesami *et al*., [35] and Violeta *et al*., [78] showed that HgCl_2_ was better than NaClO for *in vitro* disinfection. Hesami *et al*., [35] reported that although 1% HgCl_2_ for 7.5 minutes caused to the 100% disinfection, all explants exhibited necrosis by this treatment. Also, Zamir *et al*., [79] showed that by increasing immersion time of 0.05% HgCl_2_ from 5 minutes 10 minutes, the survival rate of shoot tip explants significantly decreased from 67% to 37%. Due to the high toxicity of HgCl_2_, its concentration and immersion time should be optimized for minimizing the explants’ mortality and necrosis [80]. In line with our results, Daud *et al*., [81] reported that a low concentration of HgCl_2_ (0.1%) at minimum (15 seconds) immersion time caused the highest percentage (83%) of clean and alive leaf segments. Our results also showed the negative impact of high concentration of HgCl2 at long exposure on seed livability which is in agreement with previous studies [71, 82].

The H_2_O_2_ antimicrobial effects have been discovered for years and a broad spectrum of its application has been previously recommended [83]. Several studies [84–86] have previously used H_2_O_2_ for *in vitro* sterilization. A higher level (5%) of H_2_O_2_ was reported to antagonistically affect seed germination in rape and sunflower [87]. Our results showed that 17.5% H_2_O_2_ at 15 min immersion time caused to 98.96% sterilization. Similar to our results, Hesami *et al*., [35] demonstrated that among H_2_O_2_ treatments, the highest explant viability (97.78%) and the lowest infection (2.22%) were obtained from 12% H_2_O_2_ at 15 min immersion time. Our results illustrated that in the sterilization process, the type and concentration of disinfectants and immersion time have been closely related to each other and they should be optimized together. Therefore, it is necessary to optimize the in vitro sterilization protocol for using the lowest level of disinfectants at the minimum immersion time [28, 71].

Carbon nanotubes (CNTs) are a kind of one-dimensional carbonaceous nanomaterials with the structure of cylindrical graphite [88]. CNTs are able to attach with microbial cells due to their micro size of less than 100 nm, hence, they have a wide range of biomedicinally applications [89]. CNTs may have antibacterial traits [90] and according to Ikhtiari *et al*., [91], CNT and MWCNTs increased H_2_O_2_ contents in seedlings of lettuce. Nowadays, the interaction between CNTs and different biological processes has attracted biologists’ attention [92]. Simon-Deckers *et al*., [93] reported that 100 mg/mL CNTs resulted in a 50–60% loss in the viability of bacterial cells. However, previous studies [94, 95] have reported conflicting results about the impact of antimicrobial activity of MWCNT. In the current study, two types of CNTs including MWCNT and NWCNT-Fe were used for *in vitro* sterilization of petunia seeds. The decrease in contamination rate was 31.2% and 43.7% for MWCNT and NWCNT-Fe, respectively, suggesting NWCNT-Fe may exhibit more antimicrobial activity than MWCNT under *in vitro* culture conditions. Based on the best of our knowledge, the antimicrobial activity of NWCNT-Fe in plant tissue culture systems was elucidated for the first time in the present study. Also, NWCNT-Fe for seed germination percentage was better than MWCNT. In line with our results, Arokiyaraj *et al*., [96] showed that iron oxide nanoparticles have antimicrobial activities.

A comparison of these decontaminants found that NaOCl, Ca(ClO)_2_, and H_2_O_2_ were effective in eliminating microorganisms from the surface of the explants, but their effectiveness was dependent on the concentration and exposure time. HgCl_2_ was also effective in eliminating microorganisms, but its toxicity and potential environmental hazards limit its use [35]. However, higher concentrations of HgCl_2_ led to damage to the seeds. Although CNTs have emerged as a promising decontamination agent for plant tissue culture, CNTs were not effective in eliminating microorganisms in our study. Moreover, CNTs are relatively new and their long-term environmental impact is not yet fully understood [96]. To the best of the author’s knowledge, although *in vitro* micropropagation success of different petunia cultivars have been reported in previous studies [2, 4, 5], however, this investigation is the first study about the effect of different disinfectants at various immersion time and their interactions on *in vitro* seed sterilization and germination as well as to develop efficient petunia tissue culture protocol.

Most of the *in vitro* culture processes, due to their multifactorial behavior, are difficult to understand and interpret and cannot be elucidated by traditional statistical approaches such as ANOVA, t-tests, correlation, and regression, specifically when the variables investigated are nonlinear, noisy, complex, and vague in nature [39]. The knowledge derived from ANNs, as complex mathematical algorithms, on the complex non-linear relationships of the datasets will be understandable and interpreted since that ANNs present superior prediction powers over traditional statistical methodologies to analyze complex and unpredictable variables [40]. In spite of the many advantages of ANNs, uncertainty in ML outcomes is one of the main constraints of the ML application [48]. Data quality, the sample of data collected from the domain, and model fit are the three important sources of uncertainty in ML studies [44]. Several researchers have recommended the application of different machine learning to tackle uncertainty issues [29, 43, 60, 63]. Therefore, three neural network approaches (MLP, RBF, and GRNN) were employed for modeling contamination rate and seed germination percentage on *in vitro* seed sterilization and germination of petunia. Among the three models proposed in this paper, the GRNN model performed better than the MLP and RBF models, due to its robust performance and superior predictive accuracy in training and testing subsets [50]. Additionally, our results found a similar performance between MLP and RBF as the second-best ANN models for predicting both *in vitro* parameters (i.e., contamination rate and seed germination percentage). These findings are completely in accordance with the previous finding of Hesami *et al*., [29], who has reported that the GRNN model had the best performance and accuracy in comparison to RBF and MLP models on *in vitro* seed germination of *Cannabis sativa*. There are rare investigations concerning to—use of comparative analysis of ML algorithms in the field of plant tissue culture. But several studies in other fields of biology have evidently confirmed that GRNN had better performance than MLP and RBF [29, 63, 97]. Also, according to a comparative analysis of the MLP and GRNN to optimize greenhouse banana fruit yield by Ramezanpour and Farajpour [98], their results have strongly stated that the GRNN was a more accurate technique than MLP in the prediction of evaluated parameters. In another study, the good performance of the GRNN technique for modeling and predicting *in vitro* seed germination in cannabis has been highlighted by Pepe *et al*., [28].

ANN models are difficult to interpret and use, due to they are “black box” in training and there is not any knowledge of the mathematical relationship between the input and output variables [40, 48, 64]. However, the optimization algorithms can help in the interpretation of results or the use of the models [54]. The analysis with NSGA-II algorithms based on ANNs provides the ability to answer “How to get” questions to obtain simultaneously the optimal most suitable culture medium to improve a set of desirable values for the studied parameters [39, 48, 97]. In the current research, we linked the GRNN to the NSGA-II algorithm as a computational forecasting approach to predicting and identifying critical factors that influence the *in vitro* responses (i.e., contamination rate and seed germination percentage). Successful applications of optimization algorithms, especially NSGA-II, in the field of plant tissue culture have been already accomplished [35, 39, 48, 52]. Furthermore, in agreement with our findings, the successfully results of different ANN algorithms based on different optimization algorithms have been previously carried out for modeling and predicting optimal plant tissue culture media of different species such as cannabis [63], passion fruit [50], hazel [97], and wheat [49]. This hybrid approach takes advantage of ANN’s ability to model complex non-linear relationships between input and output variables and NSGA-II’s ability to search for the optimal set of parameters [57]. The benefits of this approach include faster and more accurate optimization of tissue culture procedures, as well as the ability to generalize the optimized protocol across different plant species [48]. In addition, the use of a hybrid of ANN and NSGA-II in the optimization of plant tissue culture procedures can also help to decrease the number of treatments required for optimization [53]. Traditionally, plant tissue culture optimization involves a large number of experimental treatments, each of which involves the testing of different combinations of media components, growth regulators, and environmental conditions [50]. This can be a time-consuming and resource-intensive process, especially when working with multiple plant species [63]. By using a hybrid of ANN and NSGA-II, it is possible to reduce the number of treatments required for optimization by using the computer-generated predictions of the ANN to guide the selection of experimental treatments [63]. This approach not only saves time and resources but also reduces the likelihood of missing the optimal combination of factors by narrowing down the search space [39]. Overall, the use of a hybrid of ANN and NSGA-II in plant tissue culture optimization represents a powerful tool for streamlining and improving the efficiency of plant biotechnology research [39].

## Conclusions

*In vitro* sterilization as a multifactorial and complex process is affected by many keys interacting factors. So, to evaluate the large datasets for optimizing the petunia decontamination protocol, machine learning strategies were used as a suitable alternative to traditional statistics. Based on our results, GRNN-NSGA-II displayed a more accurate and efficacious algorithm to study petunia sterilization responses to multivariable stimuli *in vitro* and optimization. The principal motivation of the current research was to provide a reliable and robust technology based on soft computing methodology, GRNN-NSGA-II, to provide new insight into the critical factors that impact the in *vitro* seed sterilization and germination of petunia.
